# 4-[(*E*)-2-(2,4,6-Trinitro­phenyl)ethyl­idene]benzonitrile

**DOI:** 10.1107/S1600536810038584

**Published:** 2010-09-30

**Authors:** Roeland De Borger, Alain Collas, Frank Blockhuys

**Affiliations:** a Department of Chemistry, University of Antwerp, Universiteitsplein 1, B-2610 Wilrijk, Belgium

## Abstract

In the crystal of the title compound, C_15_H_8_N_4_O_6_, the mol­ecules are organized in layers due to their linkage by weak C—H⋯N hydrogen bonds. The layers are themselves inter­connected by weak C—H⋯O hydrogen bonds and π–π inter­actions [centroid–centroid distances = 3.8690 (15) and 3.9017 (16) Å]. The dihedral angle between the rings is 31.9 (1)°.

## Related literature

For related nitro­stilbenes, see: Hanson *et al.* (2005 ▶); Oehlke *et al.* (2007 ▶); Gérard & Hardy (1988 ▶). The title compound was synthesized as a new ligand for iron–phosphine complexes for use in non-linear optical (NLO) applications, see: Wenseleers *et al.* (1998 ▶); Garcia *et al.* (2001 ▶); Robalo *et al.* (2006 ▶); Garcia *et al.* (2007 ▶).
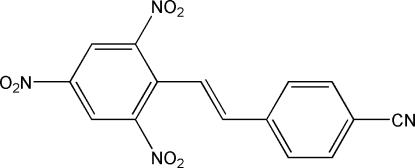


## Experimental

### 

#### Crystal data

C_15_H_8_N_4_O_6_
*M* *_r_* = 340.25
Monoclinic, 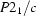

*a* = 11.183 (1) Å
*b* = 8.520 (1) Å
*c* = 15.459 (4) Å
β = 94.09 (4)°
*V* = 1469.2 (4) Å^3^
*Z* = 4
Mo *K*α radiation
μ = 0.12 mm^−1^
*T* = 293 K
0.4 × 0.4 × 0.3 mm

#### Data collection

Enraf–Nonius CAD-4 diffractometer
5370 measured reflections
2689 independent reflections
1849 reflections with *I* > 2σ(*I*)
*R*_int_ = 0.026
3 standard reflections every 60 min  intensity decay: none

#### Refinement

*R*[*F* ^2^ > 2σ(*F* ^2^)] = 0.043
*wR*(*F* ^2^) = 0.123
*S* = 1.02
2689 reflections
258 parameters
All H-atom parameters refined
Δρ_max_ = 0.17 e Å^−3^
Δρ_min_ = −0.22 e Å^−3^

### 

Data collection: *CAD-4 EXPRESS* (Enraf–Nonius, 1994 ▶); cell refinement: *CAD-4 EXPRESS*; data reduction: *XCAD4* (Harms & Wocadlo, 1996 ▶); program(s) used to solve structure: *SHELXS97* (Sheldrick, 2008 ▶); program(s) used to refine structure: *SHELXL97* (Sheldrick, 2008 ▶); molecular graphics: *ORTEP-3 for Windows* (Farrugia, 1997 ▶); software used to prepare material for publication: *WinGX* (Farrugia, 1999 ▶), *Mercury* (Macrae *et al.*, 2008 ▶) and *PLATON* (Spek, 2009 ▶).

## Supplementary Material

Crystal structure: contains datablocks I, global. DOI: 10.1107/S1600536810038584/zl2310sup1.cif

Structure factors: contains datablocks I. DOI: 10.1107/S1600536810038584/zl2310Isup2.hkl

Additional supplementary materials:  crystallographic information; 3D view; checkCIF report

## Figures and Tables

**Table 1:** Hydrogen-bond geometry (Å, °)

*D*—H⋯*A*	*D*—H	H⋯*A*	*D*⋯*A*	*D*—H⋯*A*
C3—H3⋯O32^i^	0.95 (3)	2.50 (2)	3.294 (3)	142.1 (17)
C5*A*—H5*A*⋯N1*C*^ii^	0.96 (2)	2.53 (2)	3.427 (3)	156.3 (19)
C7—H7⋯O21^iii^	0.97 (2)	2.53 (2)	3.354 (3)	143.3 (19)

Symmetry codes: (i) 
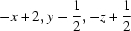
; (ii) 
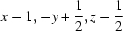
; (iii) 
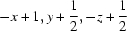
.

**Fig. 1.**
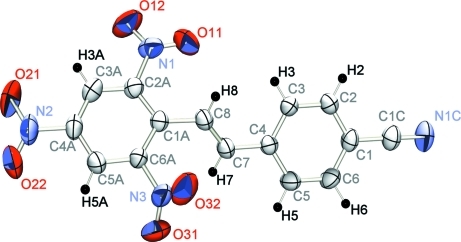
Molecular structure of the title compound showing the numbering scheme. Displacement ellipsoids are drawn at the 50% probability level; hydrogen atoms are represented by spheres with an arbitrary radius.

**Fig. 2.**
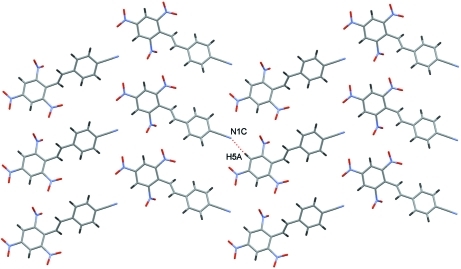
Representation of one layer of the title compound, showing the herringbone arrangement of the molecules and the associated weak hydrogen bond.

## supplementary crystallographic information

### Comment

The title compound was synthesized as a new ligand for iron-phosphine complexes for use in non-linear optical (NLO) applications [Wenseleers *et al.* (1998), Garcia *et al.* (2001), Robalo *et al.* (2006), Garcia *et al.* (2007)]. The molecular structure of the title compound (Fig. 1) is mainly determined by steric factors involving the nitro groups, which force the nitro-substituted ring out of the plane of the CH=CH fragment by 54.0 (3)°. In addition, the nitro group in the 6-position is twisted by 56.6 (3)° out of the plane of the benzene ring, whereas the nitro groups in the 2- and 4-positions remain almost in the latter plane, with torsion angles of 6.2 (3)° and 3.6 (4)°, respectively. A similar conformation of the nitro-substituted ring can be seen in other trinitrostilbenes, such as MALZOP [Hanson *et al.* (2005)], PIGBAJ [Oehlke *et al.* (2007)] and GIMBOT [Gérard & Hardy (1988)]. In the case of the latter, the two rings are parallel to each other, but the ethenylic link is rotated by approximately 90° with respect to both rings.

The fact that the nitro group in the 6-position is twisted so much more than the other two may be linked to the weak intermolecular hydrogen bond involving O32 of the nitro group and H3 of the neighbouring molecule (Table 1). As a consequence of this twist, the second oxygen atom of this nitro group, O31, comes quite close to O12 of a neighbouring molecule within the layer depicted in Fig. 2 [O31···O12^iv^, 2.821 (3) Å, symm. code iv = *x*, 1+*y*, *z*], but this should not be seen as a stabilizing contact. In fact, these layers are rather formed by the weak hydrogen bond involving H5A and the nitrogen atom N1C of the nitrile group (Table 1). The layers display a typical herringbone structure and extend along the [-1 0 2] plane. A final weak hydrogen bond, involving H7 and an oxygen atom of the nitro group in the 4-position (O21), is responsible for the organization of the molecules in the direction perpendicular to these layers (Table 1). Finally, the crystal structure displays two π–π interactions. The first involves two nitrile-substituted rings (1) of neighbouring molecules contacting each other: *Cg*(1)···*Cg*(1)^v^, 3.8690 (15) Å, 25.01°, symm. code v = 2–*x*, –y, 1–*z*. The second involves a nitrile- (1) of one and a nitro-substituted ring (2) of another molecule: *Cg*(1)···*Cg*(2)^vi^, 3.9017 (16) Å, 26.52°, symm. code vi = 2–*x*, 1/2+*y*, 1/2–*z*.

### Experimental

2,4,6-Trinitrotoluene (2.2 g, 0.0096 mol) and 4-cyanobenzaldehyde (1.3 g, 0.0096 mol) were dissolved in benzene (50 ml). Ten drops of piperidine were added, and the mixture was refluxed overnight. After cooling, the precipitate was collected by filtration. The crude product was refluxed for 4 h in *p*-xylene (25 ml) in the presence of a catalytic amount of iodine. After cooling, a mixture of yellow powder and orange-red crystals was collected, and both powder and crystals turned out to be the desired product. The yield was 12%. M.p. (uncorrected) 490–491 K. ^1^H NMR (400 MHz, CDCl_3_, TMS): δ 6.72 (d, 16.33 Hz, 1H, H7), 7.46 (d, 16.33 Hz, 1H, H8), 7.57 (d, 8.32 Hz, 2H, H2 and H6), 7.70 (d, 8.32 Hz, 2H, H3 and H5), 8.93 (s, 2H, H13 and H15) p.p.m. ^13^C NMR (100 MHz, CDCl_3_, TMS): δ 113.31 (C4), 118.24 (CN), 120.54 (C7), 122.42 (C2 and C6), 127.85 (C3 and C5), 132.78 (C13 and C15), 133.01 (C11), 135.83 (C8), 138.91 (C1), 150.32 (C14) p.p.m.

### Refinement

All the H atoms have been observed in the difference electron density map and were left to refine freely.

### Crystal data

**Table:**

C_15_H_8_N_4_O_6_	*F*(000) = 696
*M**_r_* = 340.25	*D*_x_ = 1.538 Mg m^−^^3^
Monoclinic, *P*2_1_/*c*	Melting point: 490 K
Hall symbol: -P 2ybc	Mo *K*α radiation, λ = 0.71073 Å
*a* = 11.183 (1) Å	Cell parameters from 25 reflections
*b* = 8.520 (1) Å	θ = 5.7–20.3°
*c* = 15.459 (4) Å	µ = 0.12 mm^−^^1^
β = 94.09 (4)°	*T* = 293 K
*V* = 1469.2 (4) Å^3^	Prism, orange
*Z* = 4	0.4 × 0.4 × 0.3 mm

### Data collection

**Table:**

Enraf–Nonius CAD-4 diffractometer	*R*_int_ = 0.026
Radiation source: fine-focus sealed tube	θ_max_ = 25.3°, θ_min_ = 1.8°
graphite	*h* = −13→13
non–profiled ω/2θ scans	*k* = 0→10
5370 measured reflections	*l* = −18→18
2689 independent reflections	3 standard reflections every 60 min
1849 reflections with *I* > 2σ(*I*)	intensity decay: none

### Refinement

**Table:**

Refinement on *F*^2^	Primary atom site location: structure-invariant direct methods
Least-squares matrix: full	Secondary atom site location: difference Fourier map
*R*[*F*^2^ > 2σ(*F*^2^)] = 0.043	Hydrogen site location: inferred from neighbouring sites
*wR*(*F*^2^) = 0.123	All H-atom parameters refined
*S* = 1.02	*w* = 1/[σ^2^(*F*_o_^2^) + (0.0596*P*)^2^ + 0.3738*P*] where *P* = (*F*_o_^2^ + 2*F*_c_^2^)/3
2689 reflections	(Δ/σ)_max_ < 0.001
258 parameters	Δρ_max_ = 0.17 e Å^−^^3^
0 restraints	Δρ_min_ = −0.22 e Å^−^^3^

### Special details

**Table:**

Geometry. All e.s.d.'s (except the e.s.d. in the dihedral angle between two l.s. planes) are estimated using the full covariance matrix. The cell e.s.d.'s are taken into account individually in the estimation of e.s.d.'s in distances, angles and torsion angles; correlations between e.s.d.'s in cell parameters are only used when they are defined by crystal symmetry. An approximate (isotropic) treatment of cell e.s.d.'s is used for estimating e.s.d.'s involving l.s. planes.
Refinement. Refinement of *F*^2^ against ALL reflections. The weighted *R*-factor *wR* and goodness of fit *S* are based on *F*^2^, conventional *R*-factors *R* are based on *F*, with *F* set to zero for negative *F*^2^. The threshold expression of *F*^2^ > σ(*F*^2^) is used only for calculating *R*-factors(gt) *etc*. and is not relevant to the choice of reflections for refinement. *R*-factors based on *F*^2^ are statistically about twice as large as those based on *F*, and *R*- factors based on ALL data will be even larger.

### Fractional atomic coordinates and isotropic or equivalent isotropic displacement parameters (Å^2^)

**Table:**

	*x*	*y*	*z*	*U*_iso_*/*U*_eq_	
H2	1.241 (2)	−0.019 (3)	0.4595 (14)	0.054 (6)*	
H8	0.935 (2)	−0.129 (3)	0.2627 (13)	0.053 (6)*	
H3	1.0738 (19)	−0.105 (3)	0.3753 (14)	0.056 (6)*	
H7	0.800 (2)	0.102 (3)	0.3391 (14)	0.059 (6)*	
H5A	0.530 (2)	−0.005 (3)	0.1028 (15)	0.067 (7)*	
H5	0.922 (2)	0.319 (3)	0.4044 (16)	0.073 (7)*	
H3A	0.5894 (19)	−0.444 (3)	0.1969 (14)	0.060 (6)*	
H6	1.093 (2)	0.408 (3)	0.4864 (16)	0.081 (8)*	
C3	1.07779 (17)	−0.0016 (2)	0.39786 (13)	0.0451 (5)	
C4	0.98073 (17)	0.0976 (2)	0.37989 (12)	0.0423 (4)	
C1	1.18268 (18)	0.1991 (2)	0.47949 (12)	0.0465 (5)	
C2	1.17765 (18)	0.0471 (2)	0.44706 (13)	0.0467 (5)	
C6A	0.67988 (18)	−0.0359 (2)	0.17933 (13)	0.0471 (5)	
C2A	0.71612 (18)	−0.2887 (2)	0.23434 (12)	0.0457 (5)	
C8	0.86810 (18)	−0.0745 (2)	0.27531 (13)	0.0473 (5)	
C5	0.9867 (2)	0.2489 (3)	0.41437 (14)	0.0537 (5)	
C3A	0.6117 (2)	−0.3410 (3)	0.19242 (14)	0.0535 (5)	
N1	0.7863 (2)	−0.4074 (2)	0.28671 (11)	0.0619 (5)	
C1C	1.2883 (2)	0.2523 (3)	0.52933 (14)	0.0558 (5)	
C7	0.87283 (18)	0.0455 (2)	0.32863 (13)	0.0479 (5)	
N3	0.7162 (2)	0.1266 (2)	0.16228 (15)	0.0732 (6)	
C5A	0.57307 (19)	−0.0814 (3)	0.13789 (14)	0.0546 (5)	
C1A	0.75697 (17)	−0.1337 (2)	0.22990 (12)	0.0429 (5)	
O11	0.88345 (18)	−0.3743 (2)	0.31976 (14)	0.0890 (6)	
C6	1.0866 (2)	0.2992 (3)	0.46302 (14)	0.0569 (6)	
C4A	0.54232 (17)	−0.2368 (3)	0.14446 (14)	0.0534 (5)	
O12	0.7390 (3)	−0.5339 (2)	0.29474 (15)	0.1157 (9)	
N2	0.43278 (19)	−0.2919 (3)	0.09634 (17)	0.0789 (7)	
O32	0.8093 (2)	0.1460 (2)	0.12878 (15)	0.1011 (7)	
O31	0.6471 (2)	0.2295 (2)	0.18090 (18)	0.1197 (9)	
O22	0.37527 (18)	−0.2012 (3)	0.05063 (17)	0.1101 (8)	
N1C	1.37272 (19)	0.2969 (3)	0.56717 (15)	0.0788 (7)	
O21	0.4056 (2)	−0.4286 (3)	0.10513 (19)	0.1266 (9)	

### Atomic displacement parameters (Å^2^)

**Table:**

	*U*^11^	*U*^22^	*U*^33^	*U*^12^	*U*^13^	*U*^23^
C3	0.0469 (11)	0.0378 (11)	0.0491 (11)	0.0004 (8)	−0.0065 (9)	−0.0040 (9)
C4	0.0451 (10)	0.0411 (10)	0.0397 (9)	0.0012 (8)	−0.0041 (8)	−0.0012 (8)
C1	0.0503 (11)	0.0514 (12)	0.0366 (9)	−0.0088 (9)	−0.0047 (8)	−0.0014 (8)
C2	0.0440 (11)	0.0485 (11)	0.0465 (10)	0.0030 (9)	−0.0059 (9)	0.0007 (9)
C6A	0.0514 (11)	0.0383 (10)	0.0498 (11)	−0.0012 (9)	−0.0104 (9)	−0.0035 (8)
C2A	0.0546 (12)	0.0432 (10)	0.0387 (9)	0.0006 (9)	−0.0007 (9)	−0.0016 (8)
C8	0.0420 (11)	0.0511 (12)	0.0475 (11)	0.0035 (10)	−0.0068 (9)	−0.0040 (9)
C5	0.0591 (13)	0.0443 (11)	0.0553 (12)	0.0106 (10)	−0.0119 (10)	−0.0083 (9)
C3A	0.0590 (13)	0.0495 (13)	0.0526 (12)	−0.0124 (10)	0.0072 (10)	−0.0040 (10)
N1	0.0905 (15)	0.0464 (11)	0.0474 (10)	0.0094 (10)	−0.0049 (10)	−0.0008 (8)
C1C	0.0575 (13)	0.0616 (13)	0.0471 (11)	−0.0099 (11)	−0.0046 (10)	−0.0048 (10)
C7	0.0425 (11)	0.0510 (12)	0.0484 (11)	0.0042 (9)	−0.0085 (9)	−0.0049 (9)
N3	0.0892 (16)	0.0438 (12)	0.0799 (14)	−0.0076 (11)	−0.0406 (13)	0.0026 (10)
C5A	0.0505 (12)	0.0550 (13)	0.0557 (12)	0.0062 (10)	−0.0136 (10)	−0.0084 (10)
C1A	0.0453 (10)	0.0443 (11)	0.0383 (10)	−0.0002 (8)	−0.0018 (8)	−0.0035 (8)
O11	0.0780 (13)	0.0843 (13)	0.1003 (14)	0.0135 (10)	−0.0249 (11)	0.0245 (11)
C6	0.0695 (14)	0.0422 (12)	0.0568 (12)	0.0000 (10)	−0.0104 (11)	−0.0118 (10)
C4A	0.0395 (10)	0.0625 (14)	0.0576 (12)	−0.0098 (10)	−0.0015 (9)	−0.0139 (10)
O12	0.180 (2)	0.0462 (11)	0.1130 (17)	−0.0147 (13)	−0.0469 (16)	0.0161 (10)
N2	0.0495 (12)	0.0910 (18)	0.0940 (16)	−0.0141 (12)	−0.0099 (11)	−0.0279 (14)
O32	0.1167 (18)	0.0734 (13)	0.1105 (17)	−0.0391 (12)	−0.0114 (14)	0.0248 (11)
O31	0.1395 (19)	0.0462 (11)	0.165 (2)	0.0184 (12)	−0.0491 (17)	−0.0185 (12)
O22	0.0677 (12)	0.1224 (19)	0.1324 (19)	0.0063 (12)	−0.0469 (13)	−0.0296 (15)
N1C	0.0676 (13)	0.0894 (16)	0.0764 (14)	−0.0197 (12)	−0.0161 (11)	−0.0150 (12)
O21	0.0924 (16)	0.1044 (18)	0.177 (3)	−0.0517 (14)	−0.0298 (15)	−0.0160 (17)

### Geometric parameters (Å, °)

**Table:**

C3—C2	1.370 (3)	C8—H8	0.91 (2)
C3—C4	1.389 (3)	C5—C6	1.371 (3)
C3—H3	0.95 (2)	C5—H5	0.94 (3)
C4—C5	1.395 (3)	C3A—C4A	1.363 (3)
C4—C7	1.464 (3)	C3A—H3A	0.92 (2)
C1—C6	1.381 (3)	N1—O11	1.200 (3)
C1—C2	1.388 (3)	N1—O12	1.211 (3)
C1—C1C	1.437 (3)	C1C—N1C	1.139 (3)
C2—H2	0.91 (2)	C7—H7	0.97 (2)
C6A—C5A	1.370 (3)	N3—O32	1.206 (3)
C6A—C1A	1.397 (3)	N3—O31	1.217 (3)
C6A—N3	1.472 (3)	C5A—C4A	1.374 (3)
C2A—C3A	1.369 (3)	C5A—H5A	0.96 (2)
C2A—C1A	1.401 (3)	C6—H6	1.00 (3)
C2A—N1	1.484 (3)	C4A—N2	1.464 (3)
C8—C7	1.312 (3)	N2—O22	1.202 (3)
C8—C1A	1.472 (3)	N2—O21	1.213 (3)
			
C2—C3—C4	121.39 (19)	C2A—C3A—H3A	120.2 (14)
C2—C3—H3	120.1 (13)	O11—N1—O12	123.6 (2)
C4—C3—H3	118.5 (13)	O11—N1—C2A	119.96 (19)
C3—C4—C5	118.13 (18)	O12—N1—C2A	116.4 (2)
C3—C4—C7	121.71 (18)	N1C—C1C—C1	178.3 (3)
C5—C4—C7	120.14 (18)	C8—C7—C4	124.83 (19)
C6—C1—C2	119.91 (18)	C8—C7—H7	119.5 (13)
C6—C1—C1C	120.16 (19)	C4—C7—H7	115.5 (13)
C2—C1—C1C	119.92 (19)	O32—N3—O31	125.8 (2)
C3—C2—C1	119.59 (19)	O32—N3—C6A	117.7 (2)
C3—C2—H2	121.1 (14)	O31—N3—C6A	116.5 (3)
C1—C2—H2	119.3 (13)	C6A—C5A—C4A	116.9 (2)
C5A—C6A—C1A	125.10 (19)	C6A—C5A—H5A	117.8 (14)
C5A—C6A—N3	115.15 (18)	C4A—C5A—H5A	125.1 (14)
C1A—C6A—N3	119.60 (17)	C6A—C1A—C2A	113.55 (17)
C3A—C2A—C1A	123.62 (19)	C6A—C1A—C8	121.90 (17)
C3A—C2A—N1	115.90 (18)	C2A—C1A—C8	124.53 (18)
C1A—C2A—N1	120.48 (18)	C5—C6—C1	120.1 (2)
C7—C8—C1A	124.16 (19)	C5—C6—H6	121.8 (15)
C7—C8—H8	122.3 (13)	C1—C6—H6	118.1 (15)
C1A—C8—H8	113.6 (13)	C3A—C4A—C5A	122.16 (19)
C6—C5—C4	120.9 (2)	C3A—C4A—N2	119.4 (2)
C6—C5—H5	118.5 (15)	C5A—C4A—N2	118.4 (2)
C4—C5—H5	120.7 (15)	O22—N2—O21	123.7 (2)
C4A—C3A—C2A	118.6 (2)	O22—N2—C4A	119.0 (2)
C4A—C3A—H3A	121.2 (14)	O21—N2—C4A	117.2 (3)
			
C2—C3—C4—C5	−0.8 (3)	N3—C6A—C5A—C4A	173.1 (2)
C2—C3—C4—C7	−178.91 (19)	C5A—C6A—C1A—C2A	0.9 (3)
C4—C3—C2—C1	−0.3 (3)	N3—C6A—C1A—C2A	−174.5 (2)
C6—C1—C2—C3	0.9 (3)	C5A—C6A—C1A—C8	−177.5 (2)
C1C—C1—C2—C3	−178.44 (19)	N3—C6A—C1A—C8	7.2 (3)
C3—C4—C5—C6	1.4 (3)	C3A—C2A—C1A—C6A	0.9 (3)
C7—C4—C5—C6	179.5 (2)	N1—C2A—C1A—C6A	−178.91 (18)
C1A—C2A—C3A—C4A	−0.9 (3)	C3A—C2A—C1A—C8	179.2 (2)
N1—C2A—C3A—C4A	178.93 (18)	N1—C2A—C1A—C8	−0.6 (3)
C3A—C2A—N1—O11	174.0 (2)	C7—C8—C1A—C6A	53.8 (3)
C1A—C2A—N1—O11	−6.2 (3)	C7—C8—C1A—C2A	−124.3 (2)
C3A—C2A—N1—O12	−7.4 (3)	C4—C5—C6—C1	−0.9 (3)
C1A—C2A—N1—O12	172.4 (2)	C2—C1—C6—C5	−0.3 (3)
C6—C1—C1C—N1C	−71 (9)	C1C—C1—C6—C5	179.0 (2)
C2—C1—C1C—N1C	109 (9)	C2A—C3A—C4A—C5A	−0.9 (3)
C1A—C8—C7—C4	174.55 (19)	C2A—C3A—C4A—N2	177.55 (19)
C3—C4—C7—C8	−20.7 (3)	C6A—C5A—C4A—C3A	2.4 (3)
C5—C4—C7—C8	161.3 (2)	C6A—C5A—C4A—N2	−176.0 (2)
C5A—C6A—N3—O32	−119.2 (2)	C3A—C4A—N2—O22	−176.2 (2)
C1A—C6A—N3—O32	56.6 (3)	C5A—C4A—N2—O22	2.3 (3)
C5A—C6A—N3—O31	58.5 (3)	C3A—C4A—N2—O21	3.6 (3)
C1A—C6A—N3—O31	−125.7 (2)	C5A—C4A—N2—O21	−177.9 (2)
C1A—C6A—C5A—C4A	−2.5 (3)		

### Hydrogen-bond geometry (Å, °)

**Table:**

*D*—H···*A*	*D*—H	H···*A*	*D*···*A*	*D*—H···*A*
C3—H3···O32^i^	0.95 (3)	2.50 (2)	3.294 (3)	142.1 (17)
C5A—H5A···N1C^ii^	0.96 (2)	2.53 (2)	3.427 (3)	156.3 (19)
C7—H7···O21^iii^	0.97 (2)	2.53 (2)	3.354 (3)	143.3 (19)

Symmetry codes: (i) −*x*+2, *y*−1/2, −*z*+1/2; (ii) *x*−1, −*y*+1/2, *z*−1/2; (iii) −*x*+1, *y*+1/2, −*z*+1/2.
